# Supplementary material for: Learning to detect AI texts and learning the limits
Source: PLoS One. 2025 Oct 15;20(10):e0333007. doi: 10.1371/journal.pone.0333007 (PMC12527182; doi:10.1371/journal.pone.0333007)
Supplement: S2 File — All data and scripts used in the study, some additional descriptive statistics, scalable formate figures. (ZIP) [file pone.0333007.s002.tgz › data and scripts/OSF/scripts/analysis/mixed models jamovi/outputs/questionaire_correctness_all.html]

Results


# Generalized Linear Model

| Model Info | | | | | |
| --- | --- | --- | --- | --- | --- |
| Info | | Value | | Comment | |
| Model Type |  | Linear |  | Classical Regression/ANOVA |  |
| Call |  | glm |  | Correctness ~ 1 + `Demographics: ai\_improve` + `Demographics: ai\_risk` + `Demographics: ai\_quality` + `Demographics: ai\_lacks\_creativity` + `Demographics: ai\_lacks\_understanding` + `Demographics: ai\_grammar` + `Demographics: ai\_avoidance` + `Demographics: ai\_dumbing` + `Demographics: ai\_fascination` |  |
| Link function |  | Identity |  | Coefficients in the same scale of y |  |
| Distribution |  | Gaussian |  | Normal distribution of residual |  |
| R-squared |  | 0.0636 |  | Proportion of reduction of error |  |
| AIC |  | 1304.0900 |  | Less is better |  |
| BIC |  | 1343.0010 |  | Less is better |  |
| Deviance |  | 2314.7507 |  | Less is better |  |
| Residual DF |  | 244 |  |  |  |
| Chi-squared/DF |  | 9.4867 |  | Overdispersion indicator |  |
| Converged |  | yes |  | Whether the estimation found a solution |  |
|  |  |  |  |  |  |
| --- | --- | --- | --- | --- | --- |
|  | | | | | |

## Model Results

| Loglikelihood ratio tests | | | | | | | |
| --- | --- | --- | --- | --- | --- | --- | --- |
|  | | X² | | df | | p | |
| Demographics: ai\_improve |  | 3.69709 |  | 1 |  | 0.055 |  |
| Demographics: ai\_risk |  | 7.93e-4 |  | 1 |  | 0.978 |  |
| Demographics: ai\_quality |  | 0.60676 |  | 1 |  | 0.436 |  |
| Demographics: ai\_lacks\_creativity |  | 0.01773 |  | 1 |  | 0.894 |  |
| Demographics: ai\_lacks\_understanding |  | 0.01032 |  | 1 |  | 0.919 |  |
| Demographics: ai\_grammar |  | 6.84723 |  | 1 |  | 0.009 |  |
| Demographics: ai\_avoidance |  | 0.17708 |  | 1 |  | 0.674 |  |
| Demographics: ai\_dumbing |  | 0.01796 |  | 1 |  | 0.893 |  |
| Demographics: ai\_fascination |  | 0.00247 |  | 1 |  | 0.960 |  |
|  |  |  |  |  |  |  |  |
| --- | --- | --- | --- | --- | --- | --- | --- |
|  | | | | | | | |

| Parameter Estimates | | | | | | | | | | | | | | | | | | | |
| --- | --- | --- | --- | --- | --- | --- | --- | --- | --- | --- | --- | --- | --- | --- | --- | --- | --- | --- | --- |
|  | | | | | | 95% Confidence Interval | | | |  | | 95% Exp(B) Confidence Interval | | | |  | | | |
| Names | | Estimate | | SE | | Lower | | Upper | | exp(B) | | Lower | | Upper | | z | | p | |
| (Intercept) |  | 10.20866 |  | 0.193 |  | 9.82988 |  | 10.587 |  | 27137.218 |  | 18580.728 |  | 39634.00 |  | 52.8237 |  | < .001 |  |
| Demographics: ai\_improve |  | 0.30906 |  | 0.161 |  | -0.00598 |  | 0.624 |  | 1.362 |  | 0.994 |  | 1.87 |  | 1.9228 |  | 0.056 |  |
| Demographics: ai\_risk |  | -0.00375 |  | 0.133 |  | -0.26487 |  | 0.257 |  | 0.996 |  | 0.767 |  | 1.29 |  | -0.0282 |  | 0.978 |  |
| Demographics: ai\_quality |  | 0.11801 |  | 0.152 |  | -0.17893 |  | 0.415 |  | 1.125 |  | 0.836 |  | 1.51 |  | 0.7789 |  | 0.437 |  |
| Demographics: ai\_lacks\_creativity |  | 0.01889 |  | 0.142 |  | -0.25909 |  | 0.297 |  | 1.019 |  | 0.772 |  | 1.35 |  | 0.1332 |  | 0.894 |  |
| Demographics: ai\_lacks\_understanding |  | 0.01370 |  | 0.135 |  | -0.25051 |  | 0.278 |  | 1.014 |  | 0.778 |  | 1.32 |  | 0.1016 |  | 0.919 |  |
| Demographics: ai\_grammar |  | 0.37730 |  | 0.144 |  | 0.09470 |  | 0.660 |  | 1.458 |  | 1.099 |  | 1.93 |  | 2.6167 |  | 0.009 |  |
| Demographics: ai\_avoidance |  | 0.05052 |  | 0.120 |  | -0.18477 |  | 0.286 |  | 1.052 |  | 0.831 |  | 1.33 |  | 0.4208 |  | 0.674 |  |
| Demographics: ai\_dumbing |  | -0.01658 |  | 0.124 |  | -0.25911 |  | 0.226 |  | 0.984 |  | 0.772 |  | 1.25 |  | -0.1340 |  | 0.893 |  |
| Demographics: ai\_fascination |  | -0.00864 |  | 0.174 |  | -0.34936 |  | 0.332 |  | 0.991 |  | 0.705 |  | 1.39 |  | -0.0497 |  | 0.960 |  |
|  |  |  |  |  |  |  |  |  |  |  |  |  |  |  |  |  |  |  |  |
| --- | --- | --- | --- | --- | --- | --- | --- | --- | --- | --- | --- | --- | --- | --- | --- | --- | --- | --- | --- |
|  | | | | | | | | | | | | | | | | | | | |
